# A multiplex microfluidic device to detect miRNAs for diagnosis of plant growth status

**DOI:** 10.1093/hr/uhae323

**Published:** 2024-11-26

**Authors:** Yaichi Kawakatsu, Mitsuo Hara, Ken-ichi Kurotani, Akihide Arima, Yoshinobu Baba, Michitaka Notaguchi

**Affiliations:** Bioscience and Biotechnology Center, Nagoya University, Furo-cho, Chikusa-ku, Nagoya 464-8601, Japan; Department of Molecular and Macromolecular Chemistry, Graduate School of Engineering, Nagoya University, Furo-cho, Chikusa-ku, Nagoya, Aichi 464-8603, Japan; Bioscience and Biotechnology Center, Nagoya University, Furo-cho, Chikusa-ku, Nagoya 464-8601, Japan; Institute of Nano-Life-Systems, Institutes of Innovation for Future Society, Nagoya University, Furo-cho, Chikusa-ku, Nagoya 464-8603, Japan; Institute of Nano-Life-Systems, Institutes of Innovation for Future Society, Nagoya University, Furo-cho, Chikusa-ku, Nagoya 464-8603, Japan; Department of Biomolecular Engineering, Graduate School of Engineering, Nagoya University, Furo-cho, Chikusa-ku, Nagoya 464-8603, Japan; Institute of Quantum Life Science, National Institutes for Quantum Science and Technology (QST), Anagawa 4-9-1, Inage-ku, Chiba 263-8555, Japan; Bioscience and Biotechnology Center, Nagoya University, Furo-cho, Chikusa-ku, Nagoya 464-8601, Japan; Department of Plant Production Sciences, Graduate School of Bioagricultural Sciences, Nagoya University, Furo-cho, Chikusa-ku, Nagoya 464-8601, Japan; Department of Botany, Graduate School of Science, Kyoto University, Kitashirakawa Oiwake-cho, Sakyo-ku, Kyoto 606-8502, Japan

Dear Editor, 

MicroRNAs (miRNAs) are non-protein-coding RNAs of 19–24 nucleotides that target mRNA transcripts harboring complementary sequences and regulate various environmental responses and growth in plants [1]. For instance, miR399 has been found to be upregulated in response to a phosphate deficiency in the soil and targets the transcript of the ubiquitin-conjugating E2 enzyme PHOSPHATE 2 (PHO2) to induce Pi transporters [2]. Hundreds of miRNA species are encoded in plant genome and are involved in a broad range of biological processes such as the control of flowering time, shoot maintenance, and developmental processes. To engage miRNA information in agricultural challenges, we recently developed a device to detect one type of miRNA from plant exudates prepared by brief filtering through a column without RNA purification procedures [3]. The detection system of the microfluidic devices is based on sandwich hybridization, in which biotinylated DNA binds to DNA probe immobilized in the device channel via the target miRNA, and the biotin binds to streptavidin, Alexa Fluor™ 555 conjugate (Alexa-SA), resulting in fluorescent labeling of the target miRNA semiquantitatively [3, 4]. Furthermore, the detection limit of miR399 in the system [3] was evaluated to be 0.01 nM, with a linear range of 0.01–10 nM. This system has sequence specificity, so it was expected to detect multiple miRNAs simultaneously by creating detection surfaces with different immobilized probes. In this study, we have developed the plant diagnosis system to simultaneously detect 10 miRNAs using a multiprobing microfluid device and multiple miRNA probes, each of which is highly specific to its own target miRNA.

For simultaneous detection of multi-miRNA, a DNA immobilization reactor with 10 parallel channels was created (Fig. 1A). With this reactor, *N*-hydroxysuccinimide-modified DNA was immobilized on amine-terminated glass. After removing the reactor, a microfluidic device harboring 12 channels was mounted on this DNA-immobilized glass for the diagnosis of the 12 samples (Fig. 1A). For miRNA detection, a mixture of target miRNAs and biotinylated DNA probes partially hybridized to the target miRNA was injected into the device. The target miRNA then hybridized with the DNA probe immobilized on the glass surface of the device. miRNAs that were not complementary to the immobilized DNA were not captured on the glass surface; therefore, each target miRNA was detected on each detection surface (Fig. 1A).

**Figure 1:**
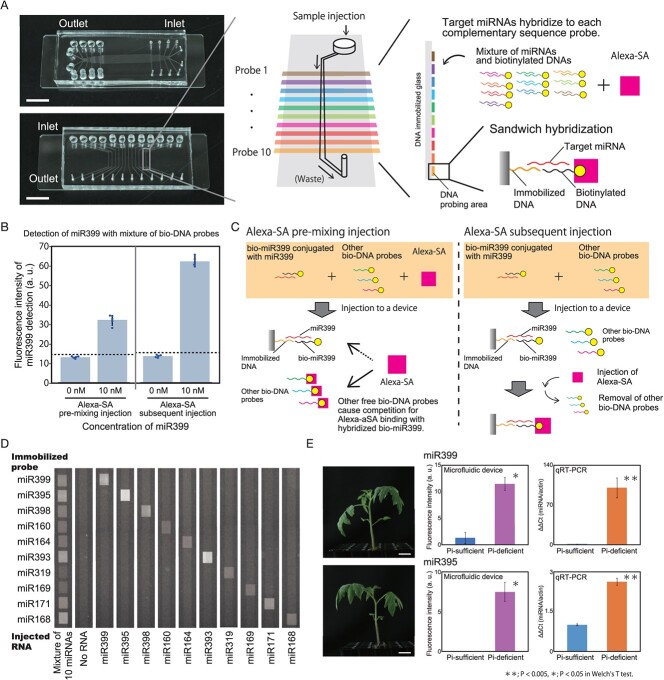
Simultaneous detection of multiple miRNAs with a microfluidic device. (A) Microfluidic devices and illustrations of the detection strategy. The device was fabricated in two steps: first, a DNA immobilization reactor with 10 horizontal channels (left upper) was mounted on a glass for the probing; second, after removal of the first reactor, a microfluidic device was mounted for miRNA detection with 12 vertical channels (left lower) on the glass. By injecting a mixture of plant exudates and a series of biotinylated DNA probes in the channel, sandwich hybridization occurred (right). (B) Detection of miR399 in the Alexa-SA premixing or subsequent injection method. Dots and dotted lines in the graph represent each data point and the signal levels at three standard deviations (SDs) above the average of 0 nM, respectively. Error bar: SD. (C) Images of signal reduction due to competition between the biotinylated DNA probe for miR399 and other biotinylated DNA probes (left). This competition can be avoided by subsequent injection of Alexa-SA (right). (D) Fluorescence detection of the 10 different varieties of plant miRNAs at 10 nM concentrations. The samples were injected from top to bottom. (E) Detection of endogenous miR399 and miR395 from the leaves of tomatoes grown under Pi-sufficient or Pi-deficient conditions. The photographs show 23-day-old tomato seedlings treated with Pi-sufficient (top) or Pi-deficient (bottom) conditions for the last 9 days. The left and right graphs show the detection of miRNAs by the microfluidic device and qRT-PCR, respectively. Scale bars: 1 cm (A) and 2 cm (E).

 Usability of the developed multidetection device was evaluated through detection of artificially synthesized tomato miR399 using a mixture of 10 different biotinylated DNAs of 0.4 μM each (bio-DNAs mixture) in two methods: Alexa-SA premixing method and Alexa-SA subsequent injection method. In the Alexa-SA premixing method, the sample liquid was added to a buffer containing bio-DNAs mixture and 0.4 μg/mL Alexa-SA and injected to the device. In the Alexa-SA subsequent injection method, sample liquid was added to a buffer containing bio-DNAs mixture and injected into the microfluidic device, followed by the injection of 4 μg/mL Alexa-SA. Using both methods, miR399 was successfully detected; however, the detection intensity was found to be stronger in the subsequent Alexa-SA injection method (Fig. 1B). This difference may be due to competition for Alexa-SA binding between the biotinylated DNA that hybridized with miR399 (bio-miR399) and other free biotinylated DNAs (Fig. 1C). In the Alexa-aSA premixing method, the detection signal was also reduced even when high concentrations of bio-miR399 were used, suggesting competition for binding to Alexa-aSA between hybridized and free biotinylated DNA. This competition was avoided using the subsequent Alexa-SA injection method.

Using the subsequent Alexa-SA injection method, simultaneous detection of 10 types of artificially synthesized miRNAs was performed. Using a DNA immobilization reactor, 10 DNA probes were immobilized in parallel and a multiple detection device was prepared. For general use, DNA probes were designed based on *Arabidopsis* miRNA sequences. Although some miRNAs are similar in sequence to each other (e.g., miR159 and miR319, or miR156 and miR157), this miRNA detection system was shown to have target specificity with fewer than two nucleotide substitutions [3]. When one type of miRNA was loaded individually, detection signals were obtained specifically on each detection surface on the channel without cross-reaction; however, when all of the 10 types of miRNAs were loaded at 10 nM concentrations, they were all detected at once (Fig. 1D). Notably, even for the detection of the same miRNA concentration, the detection signal intensities differed depending on the sequence present. The melting temperature (Tm) of the probes did not correlate with signal intensity. Secondary structure prediction of the nucleic acids using CentroidFold [5] indicated that some of the DNA probes and all of the miRNAs used in this study could form secondary structures by themselves. In addition, the biotinylated DNA probes for miR160 and miR169 could form heterodimers. In fact, the signal intensities of these miRNAs were relatively low. Taken together with the fact that the detection procedure was conducted at room temperature, secondary structures and dimer formation might be responsible for the detection signals intensity.

Using a multiplex miRNA detection device, 10 different kinds of miRNAs were detected simultaneously from tomato leaves grown under Pi-sufficient or Pi-deficient conditions to diagnose their growth condition (Fig. 1E). At the young stage, these seedlings did not show significant differences in their growth appearance. Sample preparation from tomato leaves and signal detection with a biotinylated antistreptavidin antibody (biotinylated antibody) were performed as described by Kawakatsu *et al.* (2024) [3]. Dithiothreitol (DTT) buffer was shown to prevent RNA degradation [6], and endogenous miR399 was successfully detected by Quantitative real-time PCR (qRT-PCR) from plant extracts prepared in this buffer, suggesting that DTT buffer is a suitable for simple detection of miRNAs. The total RNA concentrations in the leaf extracts ranged from 0.5 to 0.7 μg/μL. After four times of signal amplifications, the detection signal of miR399 was stronger in the Pi-deficient samples, which was confirmed by qRT-PCR (Fig. 1E). In addition, the upregulation of miR395 under Pi-deficient conditions was detected using this device and qRT-PCR (Fig. 1E). For the other miRNAs, the detection signal intensities were low, and no significant differences were identified in either the device assay or the qRT-PCR analyses. miR395 is a phloem-mediated signaling molecule induced by low sulfate levels [7]. A previous study using *Arabidopsis* showed that miR395 was downregulated in shoots under phosphorus deficiency conditions [8], which is different from the present results in tomato. This variation in miR395 expression may represent the complexity of the miRNA regulatory system and the crosstalk between different nutrient deficiency responses.

Plant diagnosis by examining biomolecules generated in plants will make it possible to detect early responses to such environmental stresses before plants exhibit phenotypic changes in their appearance. In this study, we improved a previous microfluidic device that detects one target miRNA and developed a multidetection device that detects 10 miRNA species at one time. The multidetection device could allow for rapid diagnosis of multiple symptoms in plants and increased general availability. A simple on-site diagnostic method using portable devices and kits is called point-of-care (POC) diagnostics, and microdevice-based approaches to metabolite detection were also attempted [9]. The developed device can detect miRNAs from filtered plant extracts without advanced laboratory equipment and deleterious chemicals, and it may be used for a POC diagnosis in cultivation sites. This miRNA detection system will be useful in diagnosing plant growth conditions for cultivation managements.

## Supplementary Material

Web_Material_uhae323

## Data Availability

Data generated or analyzed during this study are included in this article.

## References

[1] Zeng H, Wang G, Hu X. et al. Role of microRNAs in plant responses to nutrient stress. Plant and Soil. 2014;374:1005–21

[2] Chiou T-J, Aung K, Lin S-I. et al. Regulation of phosphate homeostasis by microRNA in *Arabidopsis*. Plant Cell. 2006;18:412–21 16387831 10.1105/tpc.105.038943 PMC1356548

[3] Kawakatsu Y, Okada R, Hara M. et al. Microfluidic device for simple diagnosis of plant growth condition by detecting miRNAs from filtered plant extracts. Plant Phenomics. 2024;6:0162 38572468 10.34133/plantphenomics.0162 PMC10988387

[4] Arata H, Komatsu H, Hosokawa K. et al. Rapid and sensitive microRNA detection with laminar flow-assisted dendritic amplification on power-free microfluidic Chip. PLoS One. 2012;7:e48329 23144864 10.1371/journal.pone.0048329 PMC3492330

[5] Sato K, Hamada M, Asai K. et al. CentroidFold: a web server for RNA secondary structure prediction. Nucleic Acids Res. 2009;37:W277–80 19435882 10.1093/nar/gkp367 PMC2703931

[6] Makoto K, Mari K, Yasuyuki N. et al. DeLTa-Seq: direct-lysate targeted RNA-Seq from crude tissue lysate. Plant Methods. 2022;18:99 35933383 10.1186/s13007-022-00930-x PMC9356424

[7] Buhtz A, Pieritz J, Springer F. et al. Phloem small RNAs, nutrient stress responses, and systemic mobility. BMC Plant Biol. 2010;10:64 20388194 10.1186/1471-2229-10-64 PMC2923538

[8] Hsieh L-C, Lin S-I, Shih AC-C. et al. Uncovering small RNA-mediated responses to phosphate deficiency in *Arabidopsis* by deep sequencing. Plant Physiol. 2009;151:2120–32 19854858 10.1104/pp.109.147280 PMC2785986

[9] Brás EJS, Margarida Fortes A, Chu V. et al. Microfluidic device for the point of need detection of a pathogen infection biomarker in grapes. Analyst. 2019;144:4871–9 31298663 10.1039/c9an01002e

